# Retained Ureteral Stent Encrustation After Stent Removal: A Case Report

**DOI:** 10.7759/cureus.44337

**Published:** 2023-08-29

**Authors:** Kalley Johnson, Lucas J Betts, Quinn Smith, Johanna Schubert

**Affiliations:** 1 Radiology, Creighton University School of Medicine, Omaha, USA

**Keywords:** ureteral stent removal, retained ureteral stent encrustation, stent encrustation, stent complications, ureteral stent

## Abstract

Ureteral stents are used to relieve acute or chronic urinary tract obstructions and may be complicated by stent encrustation. The development of encrustation is related to indwelling time, stent composition, bacterial biofilm formation, malabsorptive disorders, metabolic disorders (hypercalcemia, hyperuricosuria, pH imbalance), and cancer. Without intervention, encrustation may lead to luminal obstruction, infection, stent fracture, or ureteral avulsion during removal. Rarely, forced removal of an encrusted stent may cause the encrustation to remain in the urinary tract which can lead to further complications. Diagnosis of a retained encrustation includes evaluation with X-ray, ultrasound, and CT. Management strategies of retained encrustations are not standardized but may include removal with flexible ureteroscopy. In the following case, we present a 58-year-old male with retained encrustation material following non-forced stent removal that was not readily observed on initial imaging. CT demonstrated a curved, tubular radiodensity representing calcified encrustation material, and the diagnosis of retained encrustation was confirmed after successful removal with flexible ureteroscopy. We concluded that ureteral stent encrustation can remain in the urinary collecting system following stent removal, although this complication is rare and not well studied.

## Introduction

Ureteral stents are used to restore urinary flow from kidney to bladder in the setting of acute or chronic obstruction. Stents are often placed in the setting of ureterolithiasis to drain a ureteral obstruction, after ureterorenoscopy, or prior to extracorporeal shock wave lithotripsy or flexible ureterorenoscopy [1]. Common complications of ureteral stents include patient discomfort due to tissue irritation, irregular peristalsis, pain, infection, migration, and encrustation [2]. Encrustation occurs when minerals in the urine crystalize on the exterior or luminal surface of the stent, primarily in the form of calcium oxalate salts [3,4].

The largest risk factor for stent encrustation has repeatedly been found to be indwelling time [5]. Other risk factors for encrustation include stent composition, bacterial biofilm formation, malabsorptive disorders, metabolic disorders (hypercalcemia, hyperuricosuria, pH imbalance), and cancer. Stent encrustation may result in luminal obstruction, infection, or increase the risk of stent fracture or ureteral avulsion during removal [6]. Although a rare phenomenon, forcibly removing an encrusted stent may cause the encrustation to remain in the urinary tract or lead to ureteral injury [7]. In the following case, we present a patient with a retained ureteral stent encrustation following non-forced stent removal that was not readily observed on initial imaging. This case was previously presented at the Nebraska Radiological Society Spring Meeting and Case Competition on May 16th, 2023.

## Case presentation

**Figure 1:**
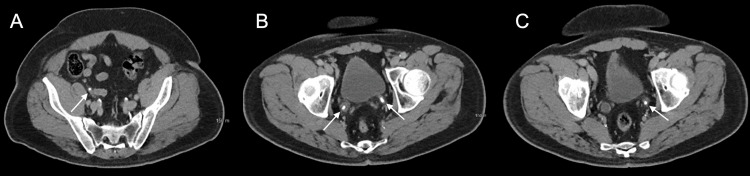
Axial images from the patient's CT abdomen and pelvis revealed four subcentimeter stones. The stones (white arrows) are seen in the right mid-ureter (A) and bilateral ureterovesicular junctions (B,C), with right greater than left distal ureteral dilation (B,C).

A 58-year-old male with a past medical history of diabetes, hypertension, and previous nephrolithiasis presented to the emergency department (ED) with a two-month history of intermittent gross, painless hematuria with slightly decreased urinary output. Vitals were within normal limits. Physical exam was significant for tenderness in the right inguinal region without evidence of trauma or hernia. There was no costovertebral angle tenderness, and the rest of the exam was unremarkable. Pertinent labs included urinalysis positive for 3+ blood, 300 mg/dL protein, and small amounts of bilirubin. A non-contrast CT scan of the abdomen and pelvis was obtained and revealed bilateral mild hydronephrosis with two obstructing subcentimeter stones in each ureter (Figure 1). Bilateral ureteroscopy with laser lithotripsy of the stones and ureteral stent placement with 6 Fr 26 cm Double-J ureteral stents was performed (day 0). Both stents were noted to be draining well during the procedure.

On postoperative day 6, the patient presented to the ED with diffuse abdominal pain, flank pain, and hematuria. Complete blood count (CBC) and basic metabolic panel (BMP) were within normal limits with no elevations in creatinine or blood urea nitrogen (BUN). Urinalysis at that time showed evidence of urinary tract infection (UTI). A non-contrast CT of the abdomen and pelvis revealed bilateral ureteric stents in place with asymmetric right periureteric fat stranding, consistent with a diagnosis of pyelonephritis. There was no evidence of stent encrustation at this time. The patient was prescribed oral cefpodoxime 100 mg twice a day for seven days for treatment of ascending UTI and naproxen for pain control. The bilateral ureteral stents were removed without excess force or difficulty on day 12 and were fully intact.

**Figure 2:**
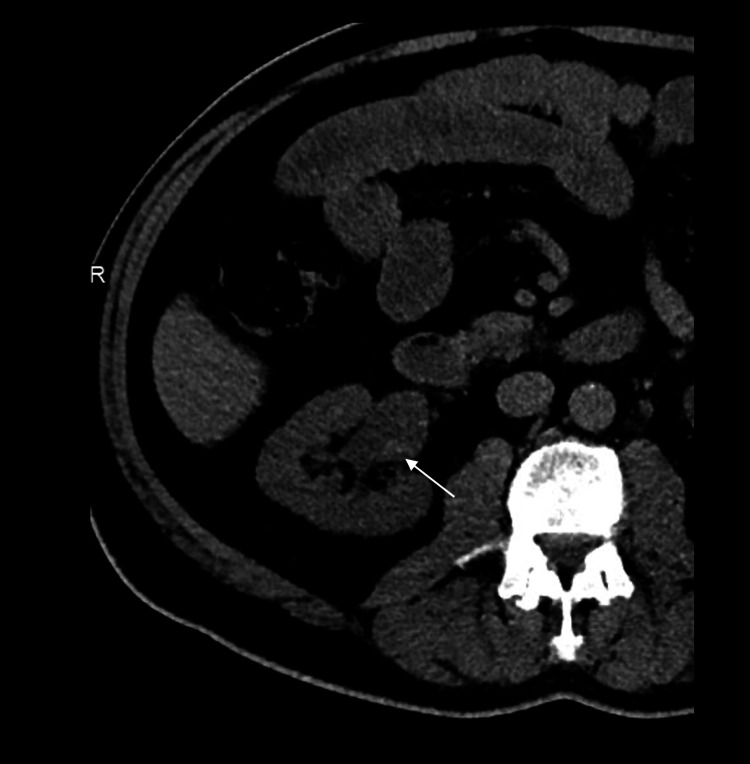
Axial image from the patient’s non-contrast CT revealed interval stent removal and right renal pelvis dilation (white arrow). A subtle area of dependent increased attenuation was noticed retrospectively in the right renal pelvis.

On postoperative day 15, the patient returned to the ED with back pain that was suspected to be musculoskeletal in nature. A non-contrast CT scan of the abdomen and pelvis showed interval stent removal with right renal pelvis dilation. In retrospect, a subtle area of dependent increased attenuation was evident in the right renal pelvis (Figure 2).

**Figure 3:**
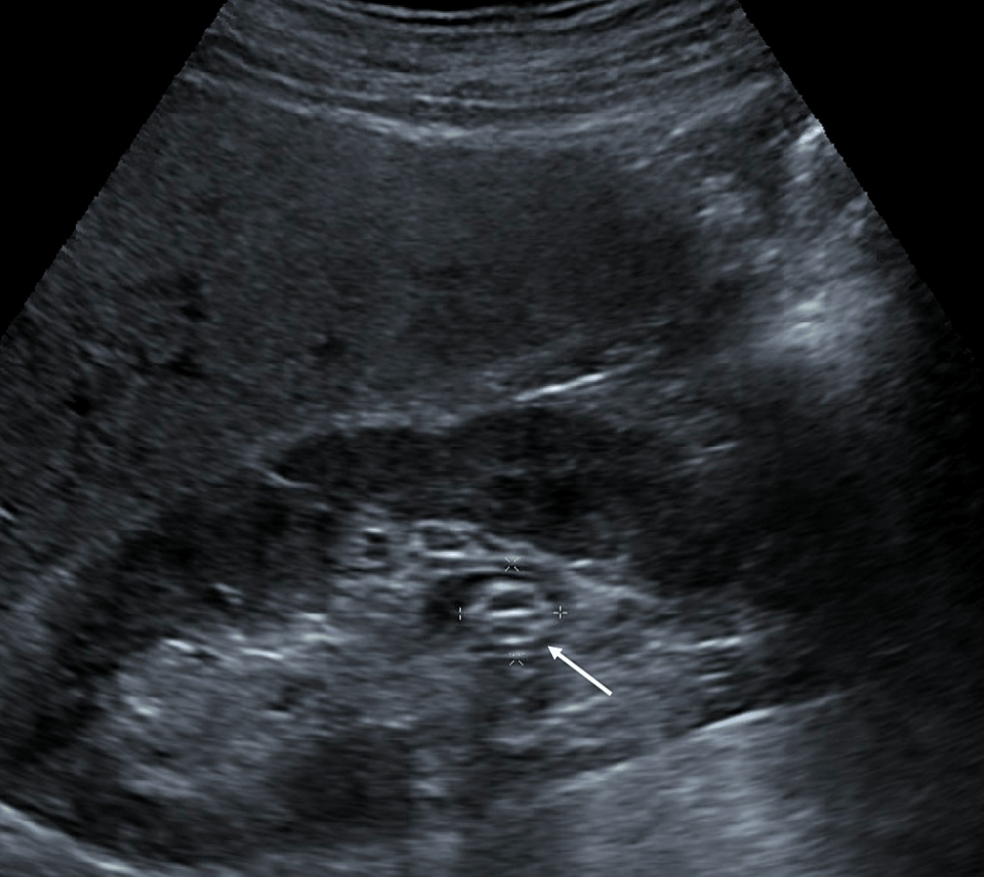
Longitudinal ultrasound image through the right kidney revealed an indeterminate echogenic mass in the right renal pelvis measuring 1.5 cm (white arrow), concerning for recurrent stone, thrombosis, hematoma, or retained encrustation material.

On postoperative day 39, the patient called his urologist complaining of back pain and continued hematuria. A renal ultrasound was performed and demonstrated an indeterminate echogenic mass in the right renal pelvis measuring 1.5 cm with no associated hydronephrosis, concerning for recurrent stone, thrombosis, hematoma, or retained encrustation material from the prior stent (Figure 3).

**Figure 4:**
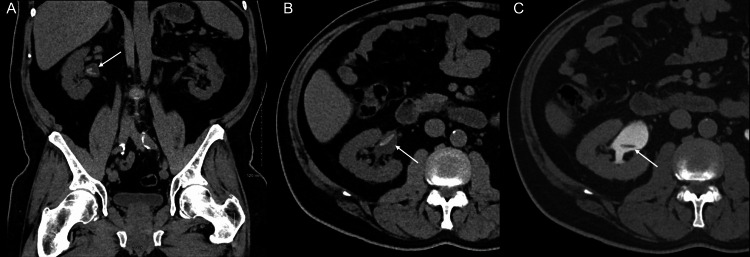
Coronal (A) and axial (B) non-contrast CT images in soft tissue window through the right kidney revealed a 2.6 x 0.5 cm tubular-shaped calcification within the right renal pelvis (white arrow). Axial delayed contrast CT (C) also through the right mid kidney demonstrated a corresponding dependent filling defect in the contrast filled renal pelvis (white arrow).

A CT of the abdomen and pelvis with and without contrast was obtained which revealed a curved, tubular radiodensity likely representing calcified encrustation material in the right renal pelvis. The encrustation measured 2.6 x 0.5 cm and appeared to be mobile within the dependent portion of the renal pelvis (Figure 4). The curvilinear shape of the calcification and preceding ureteral stent history strongly favored encrustation as the diagnosis.

Urology performed flexible ureteroscopy to remove the mass and confirmed the diagnosis of retained calcified ureteral stent encrustation material with no evidence of stent fragment present from the prior stent. The patient experienced no further complications.

## Discussion

Although the use of ureteral stents is necessary for the treatment of urinary tract obstruction, their long-standing placement brings about many consequences. Ureteral stents can serve as a nidus for bacterial colonization, biofilm formation, and crystallization, all of which can promote the development of UTI and the formation of encrustations [1,3]. Given that the encrustation was not clearly visible on CT three days post stent removal, it is possible that the encrustation developed and calcified over time on a retained biofilm.

The basis of treatment for an encrusted ureteral stent depends on the size and location of the encrustation. If the stent is still in place, it is important to understand which portion of the stent is encrusted [6]. Due to the radio-opaque composition of ureteral stent encrustation, standard kidney, ureter and bladder (KUB) X-ray is suggested as the first line to identify an encrustation. However, this is often replaced with or followed by ultrasound or CT scan to further determine the exact positioning of the encrustation relative to the ureter and/or the ureteral stent [6]. After determining the extent of encrustation, one of several grading systems is used to estimate the complexity of encrustation removal. A corresponding treatment algorithm is then utilized which ultimately helps to determine the recommended surgical technique best suited for successful removal of the encrustation [2,6]. 

The Forgotten, Encrusted, Calcified (FEcal) System classifies ureteral stent encrustation into five categories. Grade I encrustation is minimal involvement of the distal portion of the stent pigtail whereas grade V is circumferential encrustation encasing the entire stent. The authors suggest an associated algorithm with treatment ranging from cystoscopy and stent removal for minor encrustations to extracorporeal shock wave lithotripsy (ESWL), percutaneous nephrolithotomy (PCNL), cystolitholapaxy, ureteroscopy or a combination of the above for more extensive encrustations. If a patient exhibits signs of <20% renal function, nephrectomy should be considered [8].

The KUB System scores the encrustation based on severity and specific location within the urinary tract. The system grades the extent of encrustation from 1 (minor) to 5 (severe) for each of the following locations: proximal renal coil “K,” the ureteral shaft “U,” and the distal bladder coil “B” for a cumulative KUB score with a maximum score of 15 [6,9]. A treatment algorithm that integrates the FECal System with the KUB System has recently been published in the literature [6]. 

The above case presents a unique scenario where the ureteral stent encrustation was identified following stent removal. Because in this example there is no way to classify the encrustation relative to an indwelling stent, the FECal and KUB Systems and their corresponding treatment algorithms are not applicable. At this time, few cases in the literature exist that describe retained ureteral stent encrustation after removal [7]. There is currently no standardized diagnostic workup or treatment algorithm for a retained encrustation without a stent in place. In our case, flexible ureteroscopy was sufficient to remove the calcified encrustation and confirm the diagnosis. If ureteroscopy failed, a stepwise treatment approach with techniques of increasing invasiveness could be pursued with ESWL, PCNL, and open surgery.

## Conclusions

Our case indicates that a ureteral stent encrustation can remain in the urinary collecting system following stent removal. Although a rare complication, retained stent encrustation should be included in the differential diagnosis in patients presenting with persistent hematuria and back pain after ureteral stent removal. Radiologists need to have diagnostic awareness about this phenomenon, as a curvilinear hyperdense structure in the collecting system in a patient with recent ureteral stent removal may represent a retained stent encrustation that may require surgical extraction if causing complications. Further research on this topic should be conducted with the goal of standardizing and optimizing the diagnosis and treatment of retained encrustations.
